# Management of Large-Vessel Vasculitis With FDG-PET: A Systematic Literature Review and Meta-Analysis

**DOI:** 10.1097/MD.0000000000000622

**Published:** 2015-04-10

**Authors:** Michael Soussan, Patrick Nicolas, Catherine Schramm, Sandrine Katsahian, Gabriel Pop, Olivier Fain, Arsene Mekinian

**Affiliations:** From the Paris 13 University, Sorbonne Paris Cité (MS); AP-HP, Avicenne Hospital, Department of Nuclear Medicine (MS, GP); AP-HP, Avicenne Hospital, Department of Pharmacology (PN); Inserm U1138, Centre de Recherche des Cordeliers, Equipe 22, Paris 5, Paris 6 (CS, SK); APHP, HEGP, Department of Biostatistics (SK); and DHU I2P, AP-HP, Saint-Antoine Hospital, Department of Internal Medicine, Paris, France (OF, AM).

## Abstract

We aimed to clarify the role of ^18^F-fluorodeoxyglucose positron emission tomography (FDG-PET) in the management of large-vessel vasculitis (LVV), focusing on 3 issues which are as follows: describe and determine the different FDG-PET criteria for the diagnosis of vascular inflammation, the performance of FDG-PET for the diagnosis of large-vessel inflammation in giant cell arteritis (GCA) patients, and the performance of FDG-PET to evaluate the disease inflammatory activity in Takayasu arteritis (TA) patients.

MEDLINE, Cochrane Library, and EMBASE database were searched for articles that evaluated the value of FDG-PET in LVV, from January 2000 to December 2013. Inclusion criteria were American College of Rheumatology criteria for GCA or TA, definition PET positivity threshold, and >4 cases included. Sensitivity (Se) and specificity (Sp) of FDG-PET for the diagnosis of large-vessel inflammation were calculated from each included individual study, and then pooled for meta-analysis with a random-effects model.

Twenty-one studies (413 patients, 299 controls) were included in the systematic review. FDG-PET showed FDG vascular uptake in 70% (288/413) of patients and 7% (22/299) of controls. Only vascular uptake equal to or higher than the liver uptake was significantly different between GCA/TA patients and controls (*P* < 0.001). The meta-analysis of GCA patients (4 studies, 57 patients) shows that FDG-PET has high Se and Sp for the diagnosis of large-vessel inflammation in GCA patients in comparison to controls, with a pooled Se at 90% (95% confidence interval [CI], 79%–93%) and a pooled Sp at 98% (95% CI, 94%–99%). The meta-analysis of TA patients (7 studies, 191 patients) shows that FDG-PET has a pooled Se at 87% (95% CI, 78%–93%) and Sp at 73% (95% CI, 63%–81%) for the assessment of disease activity in TA, with up to 84% Sp, with studies using National Institutes of Health criteria as the disease activity assessment scale.

FDG-PET showed good performances in the diagnosis of large-vessel inflammation, with higher accuracy in GCA patients than in TA patients. Although a vascular uptake equal to or higher than the liver uptake appears to be a good criterion for the diagnosis of vascular inflammation, further studies are needed to define the threshold of significance as well as the clinical significance of the vascular uptake.

## INTRODUCTION

Large-vessel vasculitis (LVV) comprises non-necrotizing granulomatous vasculitis, with mainly 2 separate conditions: giant cell arteritis (GCA) and Takayasu arteritis (TA).^1^ Although these 2 conditions are characterized by the inflammation of aorta and its main branches, several substantial differences exist in clinical practice. For GCA, the diagnosis of large-vessel inflammation could be challenging in patients with systemic constitutional signs, unexplained inflammatory syndrome, or negative temporal artery biopsy (TAB). In patients with early-stage TA, the diagnosis of vascular inflammation with conventional imaging could be challenging, whereas in late-stage TA the persistent underlying inflammation could be difficult to assess and a reliable tool for this task is still lacking.

^18^F-fluorodeoxyglucose positron emission tomography (FDG-PET) is a hybrid imaging method with growing interest for the diagnosis of various inflammatory conditions such as sarcoidosis, vasculitis, and infections.^2–4^ Inflammatory cells such as macrophages or granulation tissue have been shown to avidly take up FDG, especially under activated conditions.^5^ Several studies assessed the interest of FDG-PET for the diagnosis of LVV and the correlation to the disease activity.^6,7^ Authors stated that FDG-PET is highly effective in assessing the disease activity and the extent of LVV. Yet, the conclusions of these studies are biased by the inclusion of small number of patients with various inclusion criteria and even polymyalgia rheumatica without GCA.^6,8,9^ In addition, no definite consensual criteria exist to define the presence of vascular inflammation by FDG-PET in LVV and different parameters such as visual or semiquantitative methods have been reported. Regarding the assessment of persistent vascular inflammation in TA, PET studies used various definitions of clinical disease activity, and large studies are still lacking.

In this systematic review and meta-analysis, we aimed to clarify the role of FDG-PET in the management of GCA and TA patients, focusing our research on 3 issues: describe and determine the different FDG-PET criteria for the diagnosis of vascular inflammation, the performance of FDG-PET for the diagnosis of large-vessel inflammation in GCA patients, and the performance of FDG-PET to evaluate the disease inflammatory activity in TA patients.

## METHODS

### Data Sources and Searches

A quantitative systematic review covering the period from January 2000 to December 2013 was undertaken by a comprehensive search of English and French medical literature with use of PubMed (MEDLINE), Web of science, and EMBASE database to identify original articles on the evaluation of FDG-PET in patients with GCA and TA. The literature search was conducted by AM and MS using the following terms: positron emission tomography, PET, FDG-PET, PET/CT, PET-CT, vasculitis, aortitis, giant cell arteritis, Horton disease, Takayasu disease, Takayasu arteritis, large-vessel vasculitis and large-vessel arteritis. Ethical approval was not necessary, as this study was based on published data.

### Study Selection

To be included in the study, the published articles had to fulfill the following criteria for GCA: American College of Rheumatology (ACR) diagnostic criteria and/or positive TAB and for TA: ACR diagnostic criteria and/or Ishikawa modified Sharma criteria^10^; description of FDG-PET positivity criteria; and >4 included patients. To be eventually included in the quantitative meta-analysis, each study had to report the complete 2 × 2 contingency table or give full data to build it. All articles were independently reviewed by AM and MS, first separately and then together to verify the inclusion criteria.

### Data Extraction

The 2 co-authors (AM and MS) reviewed together the selected articles to extract the following data: first author, year of publication, number of patients and controls, controls underlying disease, study design, geographic provenance of the study population, age, sex ratio, acute-phase reactants (erythrocyte sedimentation rate [ESR] and C-reactive protein), disease activity assessment (TA), disease activity status, and treatments before FDG-PET or time from treatment to PET realization. For TA, the criteria used for the assessment of disease activity were specified: National Institutes of Health (NIH) scale or other activities assessment.^11^ PET data were analyzed as follows: number of positive PET (using authors’ criteria); type of PET criteria for the diagnosis of vasculitis: visual analysis or semiquantitative analysis (maximum standardized uptake value [SUV_max_] or SUV_max_ normalized by standardized uptake value [SUV] of liver or inferior vena cava [IVC]); and uptake threshold.

Studies with TA providing individual data regarding clinical activity, acute phase reactants (ESR and/or C-reactive protein), PET visual grade, and SUV_max_ were identified. These individual data were pooled in order to study the relationship between clinical/biological data and PET results.

### Data Synthesis and Analysis

Descriptive statistics included the mean (standard deviation) for continuous variables and frequencies (percentages) for categorical variables. For each included study in the meta-analysis, we extracted or built the 2 × 2 table of true-positive, false-positive, true-negative, and false-negative results in order to calculate test sensitivity (Se) and specificity (Sp). When any observed count was zero, we used a continuity correction of 0.5, applied to all 4 cells of the study. The PET imaging was the index test while the diagnostic criteria of the disease (GCA or TA) or scales of disease activity for TA were considered as the reference diagnostic tests. Then, we performed univariate meta-analyses of Se and Sp to synthesize logit-transformed Se and Sp values separately, testing for homogeneity with a χ^2^ test. In addition, likelihood ratios (LRs) and diagnostic odds ratios were pooled by the DerSimonian method (random-effects model). The software Meta-DiSc version 1.4 was used to performed this meta-analysis technique.^12^ In addition to this meta-analysis, additional statistical comparisons between accordingly continuous or qualitative variables were made with JMP version 10.0 (SAS Institute Inc, Cary, NC). All *P* values were 2 tailed, and *P* < 0.05 was considered to reflect a statistical significance.

## RESULTS

### Literature Search and Analysis

The literature search yielded 264 citations of PET and PET/computed tomography (CT) in LVV, of which 106 studies corresponding to original articles were assessed for eligibility. Twenty-one studies were included in the systematic review. Among the 21 studies, 4 were included in the first meta-analysis to determine the value of FDG-PET for the diagnosis of large-vessel vascular inflammation in GCA, and 7 were included in the second meta-analysis to determine the value of FDG-PET to detect the vascular inflammation in TA with active disease (see Flow Diagram).

The systematic review (21 studies) provided 413 cases of LVV, including 127 cases of GCA (8 studies), 197 cases of TA (8 studies), and 89 cases with LVV (5 studies) that did not provide sufficient data to discriminate between GCA and TA.^13–17^ The control group included 299 patients: 226 were oncologic patients, 31 suffering from other conditions (infections n = 18, rheumatoid arthritis n = 6, and small vessel vasculitis n = 5), and 44 were undefined. At the time of PET study, 38% (156/413) of patients were under corticosteroid and/or immunosuppressive treatment.

The parameter used to detect the vascular inflammation with FDG-PET and the thresholds used to define the presence of vascular inflammation were different among the studies (Table 1). Visual analysis was used in 19/21 studies, with mostly the liver uptake used as the reference (17/19 studies), and threshold for the presence of vascular inflammation defined as slight vascular uptake (<liver uptake) in 6/17 studies, moderate uptake (=liver uptake) in 9/17 studies, or intense uptake (>liver uptake) in 2/17 studies (Table 1). The semiquantitative analysis was used in 2/21 studies to define the presence of LVV: SUV_max_ or SUV_max_ normalized by SUV of liver or IVC.

**TABLE 1:**
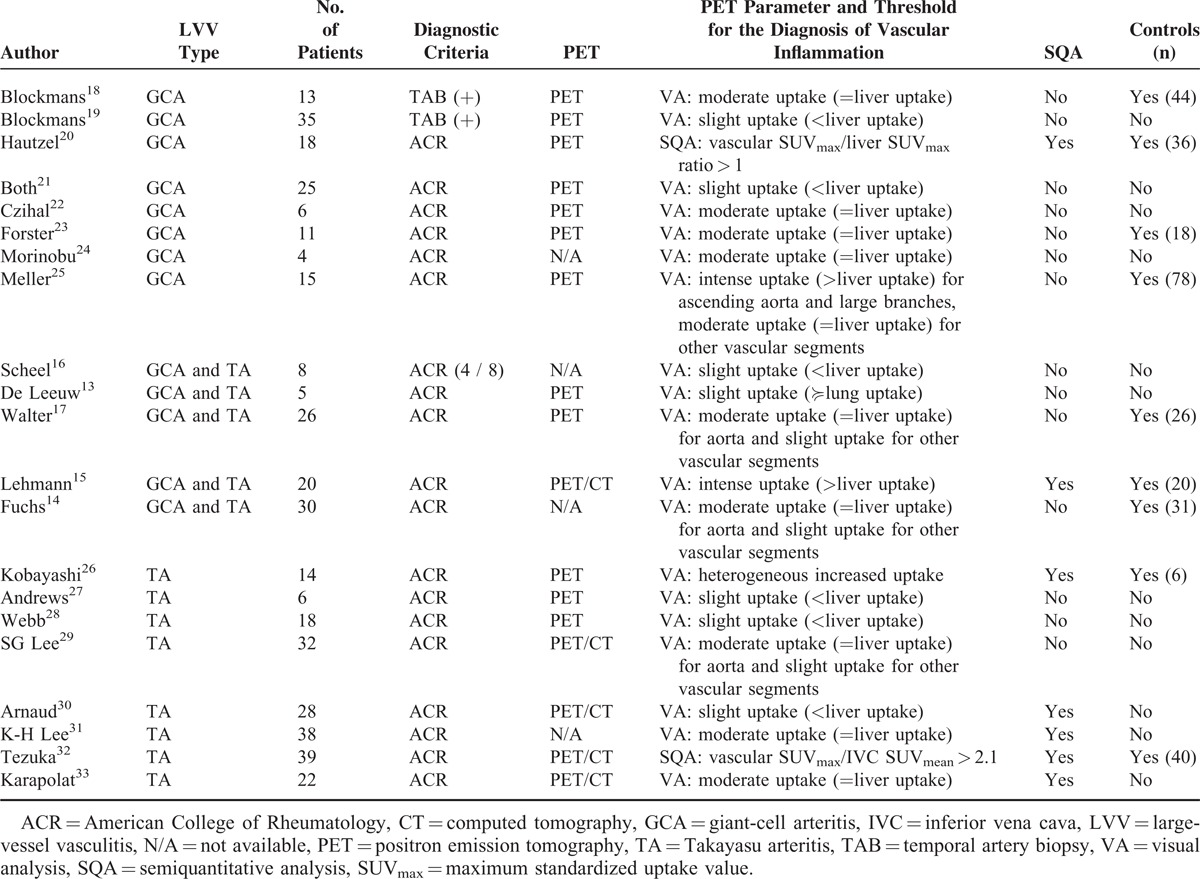
Studies Included in the Literature Review and Meta-Analysis: Descriptive Characteristics and Details of the Inclusion Criteria

### Descriptive Analysis of FDG-PET in GCA/TA: Systematic Review

FDG-PET detected a vascular arterial uptake in 70% (288/413) of patients with GCA/TA and 7% (22/299) of controls (*P* < 0.05) (Table 2). The detection of vascular arterial uptake was more frequent in GCA patients than in TA patients(110/127 = 87% vs 114/197 = 58%, respectively, *P* < 0.0001).

**TABLE 2:**
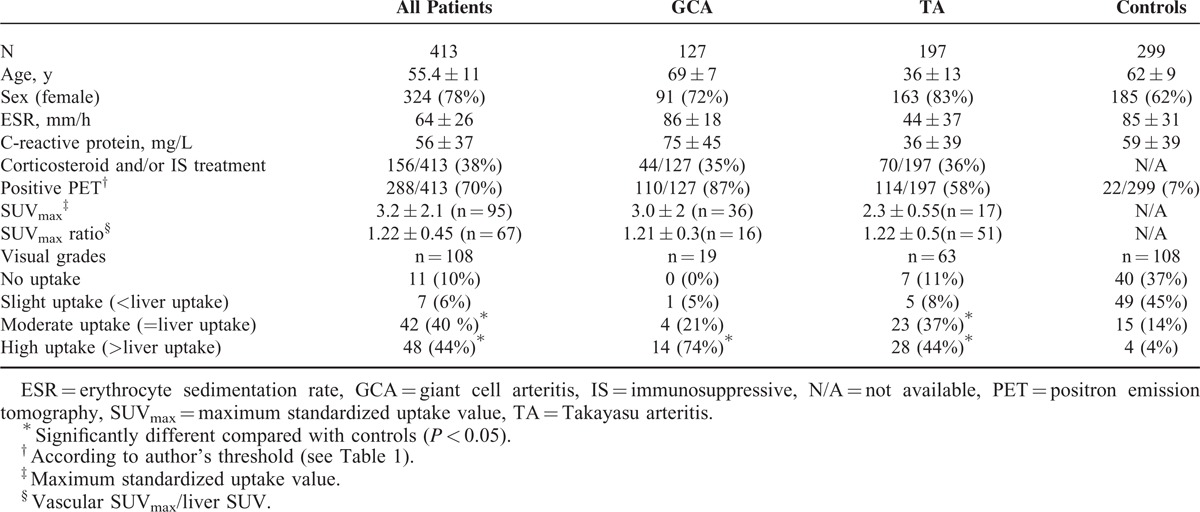
Characteristics of Patients With LVV, GCA, TA, and Controls Included in the Systematic Review

Considering the visual analysis using the liver as a reference, only moderate and high uptakes (≥ liver uptake) were significantly more frequent in GCA/TA patients than in controls (84% vs 18%, *P* < 0.001, Table 2). As much as 45% of controls exhibited a slight uptake (<liver uptake) suggesting that it should not be considered as pathological. Considering the semiquantitative analysis, the mean pooled SUV_max_ and SUV_max_ ratio (vascular SUV_max_/liver SUV) in all GCA/TA patients were 3.2 ± 2.1 and 1.22 ± 0.45, respectively. There was no available data for controls regarding SUV_max_, precluding any comparison between LVV and controls.

### FDG-PET for the Diagnosis of Large-Vessel Inflammation in GCA

**FIGURE 1:**
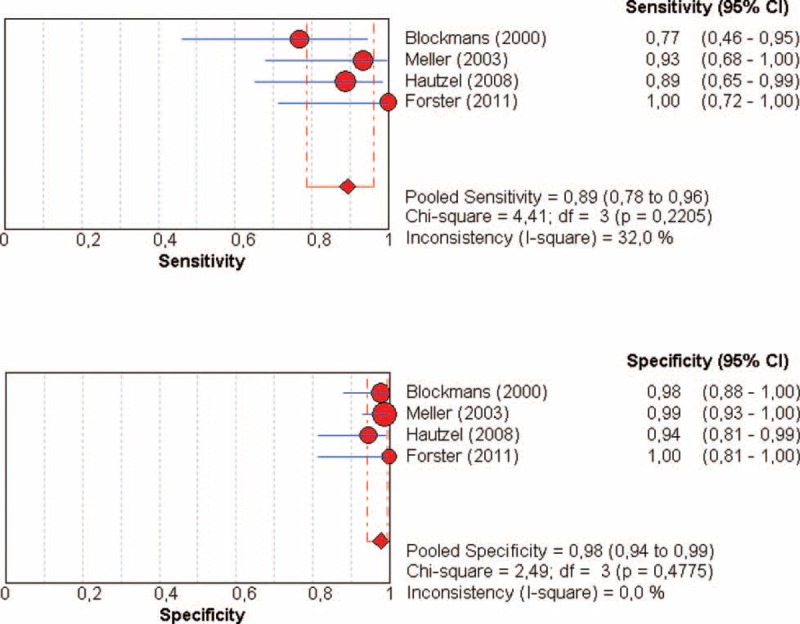
Diagnosis of LVV in patients with GCA by using FDG-PET: forest plots of eligible studies show individual and pooled sensitivities and specificities of the studies included in the meta-analysis and the related inconsistency index. FDG-PET = ^18^F-fluorodeoxyglucose positron emission tomography, GCA = giant cell arteritis, LVV = large-vessel vasculitis.

Four studies were included in this meta-analysis, with 57 patients with GCA and 176 controls (Figure 1, Table 3). FDG-PET parameter for the diagnosis of large-vessel inflammation was a visual analysis in 3 studies [threshold defined as a moderate uptake (n = 2) or intense uptake (n = 1)], or a semiquantitative analysis in 1 study (SUV_max_/liver SUV_max_ > 1). Compared with controls, the pooled Se of FDG-PET to detect the large-vessel vascular inflammation was 89.5% (95% confidence interval [CI], 78.5–96.0) with pooled Sp at 97.7% (95% CI, 94–99), with a very high positive LR (28.7). There was no significant heterogeneity or inconsistency (Figure 1, Table 3).

**TABLE 3:**
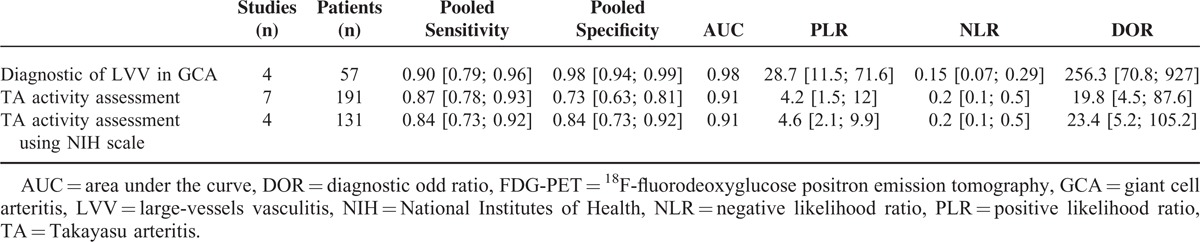
Pooled Sensitivities and Specificities of the Meta-Analysis of the Values of FDG-PET for the Diagnosis of LVV in GCA and Disease Activity Assessment in TA

### FDG-PET for the Assessment of Large-Vessel Inflammation in TA

**FIGURE 2:**
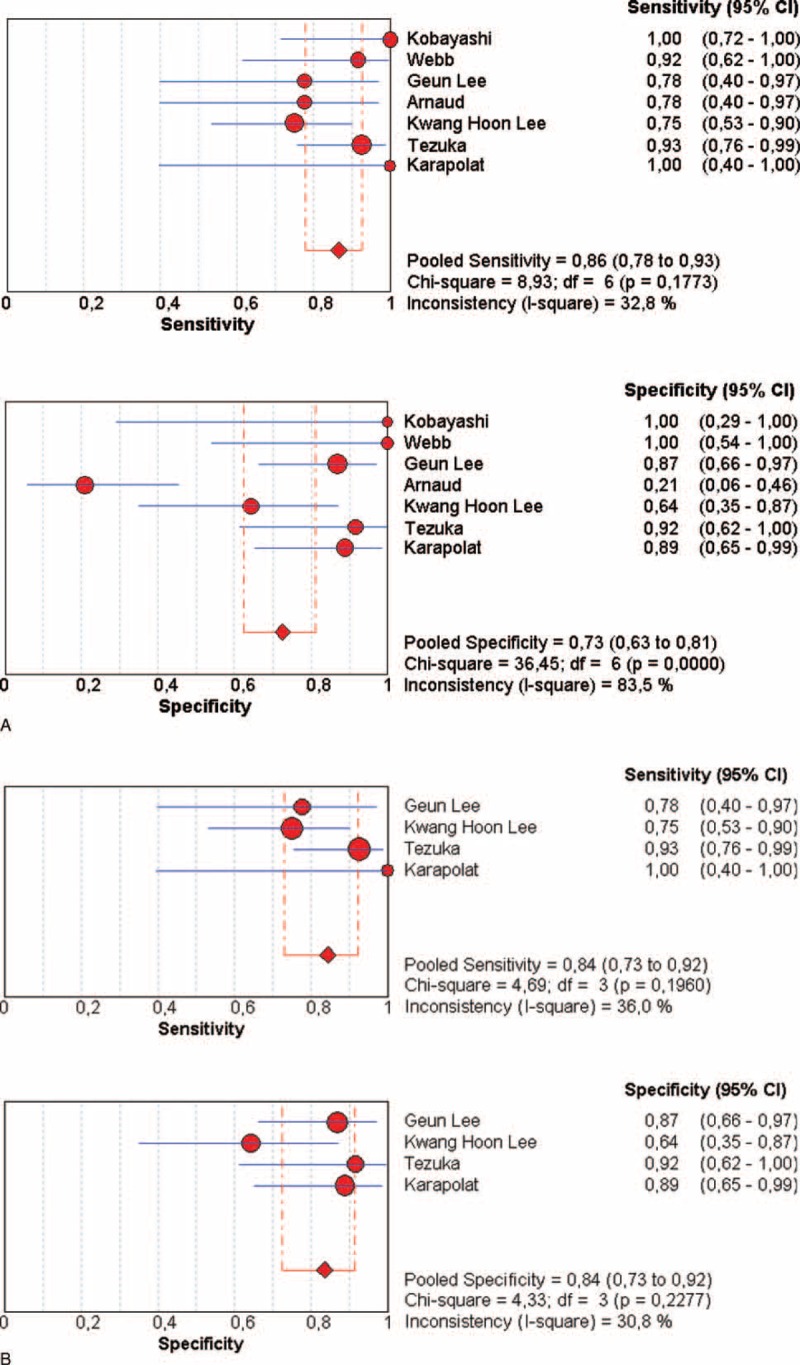
FDG-PET for the disease activity in TA: forest plots of eligible studies show individual and pooled sensitivities and specificities of the studies included in the meta-analysis: (A): all studies with TA (B): studies with disease activity evaluated only with NIH scale. Inconsistency index squared was also determined. FDG-PET = ^18^F-fluorodeoxyglucose positron emission tomography, NIH = National Institutes of Health, TA = Takayasu arteritis.

Seven studies were included in this second meta-analysis, including 191 patients with TA (N = 96 with active TA). The FDG-PET parameters used to detect the vascular inflammation were the visual analysis (n = 6 studies) and a semiquantitative analysis (n = 1) (Table 2). For the detection of large-vessel inflammation in TA according to the disease activity status (TA active/inactive disease as defined using NIH scale [n = 4 studies] or other [n = 3]), FDG-PET showed a pooled Se at 87% (95% CI, 78.0–92.6) and pooled Sp at 73% (95% CI, 62.5–81.3), with a positive LR at 4.2, and significant heterogeneity and inconsistency (I^2^ = 83.5%) (Figure 2 and Table 3). To explore the source of heterogeneity, we performed a subgroup analysis by combining the 4 studies with disease activity assessment based on NIH scale. The pooled Se remained similar at 84.4% (95% CI, 73.1–92.2) without heterogeneity (*P* = 0.196, I^2^ = 36%), and with an increased Sp at 84% (95% CI, 72.5–91.5%) (*P* = 0.134, I^2^ = 46.3%).

### Correlation of FDG Vascular Uptake With Disease Activity in TA

Individual data of 78 patients with TA were extracted from 4 studies.^30–33^ Visual analysis was used for the detection of vascular inflammation in all studies, in addition to a semiquantitative analysis in 2/4 studies (Table 4). Visual grading correlated with the presence of clinical activity (*P* = 0.01), C-reactive protein (*P* = 0.04), and ESR levels (*P* = 0.04) (Table 4). SUV_max_ ratio increased with the number of markers of disease activity (*P* = 0.01), and correlated with the presence of clinical activity (*P* = 0.01), ESR (*P* = 0.02), and C-reactive protein levels (*P* = 0.0006). Visual analysis showed that high uptake was well correlated with the presence of markers of activity: frequency of 8%, 21%, 56%, and 69% in the presence of 0, 1, 2, or 3 markers of activity. Interestingly, a significant vascular uptake (moderate and high uptakes) was observed in 67% (8/12) of TA patients without markers of activity versus 88% (14/16) in patients with the 3 markers of activity.

**TABLE 4:**
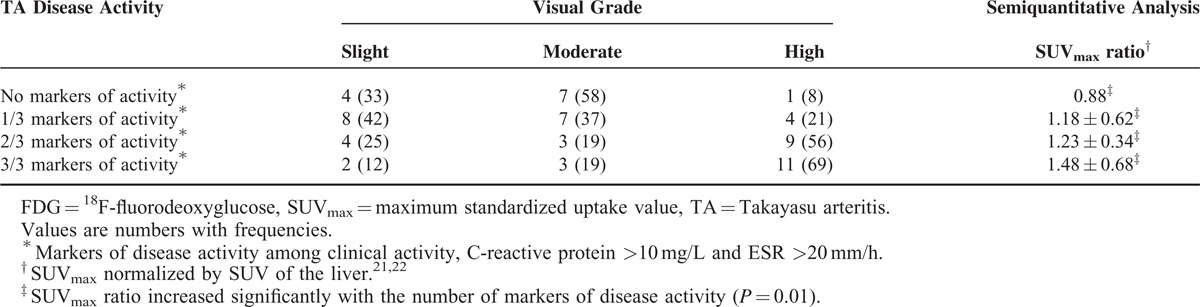
FDG Uptake Evaluated by Visual or Semiquantitative Analysis and Clinical and Biological Activity Criteria in 78 Patients With TA

## DISCUSSION

This study shows that FDG-PET has a good Se and Sp for the diagnosis of large-vessel inflammation in GCA patients and in TA patients. The performance of the FDG-PET is better in GCA patients than in TA patients (Se/Sp 90%/98% vs 84%/84%, respectively), with a higher positive LR (28.7 vs 4.2, respectively). This higher accuracy of PET in GCA could be explained by the inclusion of patients with GCA together with TA patients with various degrees of vascular inflammation.

These results are in line with previous reports, especially 1 previous meta-analysis including GCA patients, showing pooled Se and Sp at 80% and 89%, respectively.^6^ The better performance of PET for the diagnosis of large-vessel inflammation in GCA found in our study can be explained by the inclusion of patients with ACR-positive GCA and with precise definition of PET positivity. One recent study showed a Se of 80% and a Sp of 79% for the diagnosis of large vessel inflammation in a case series of biopsy-proven GCA patients, using a semiquantitative analysis (mean of the SUV_max_ at all the vascular territories).^34^

Our results provide interesting data about the definition of vascular inflammation with FDG-PET. In GCA patients, a vascular uptake, equal or higher than the liver uptake, appears as the best criteria for the detection of vascular inflammation compared with controls. On the contrary, although our results showed a correlation between the intensity of FDG uptake and markers of activity in TA, a significant FDG vascular uptake can be observed in TA patients without markers of activity. Unfortunately, there is not enough literature data to compare the value of visual analysis versus semiquantitative analysis for the detection of vascular inflammation.

The aim of this study was to assess the value of FDG-PET in the management of both GCA and TA patients. The first issue is the diagnosis of large-vessel inflammation in confirmed GCA, because of the absence of validated imaging tool and the variable rate of large-vessel inflammation according to different studies.^35^ For this purpose, we included only studies with validated criteria for the diagnosis of GCA, and particularly, excluding patients with polymyalgia rheumatica without a confirmed diagnosis of associated GCA. Only studies with a precise description of PET parameters for the diagnosis of vascular inflammation were included, and studies which did not provide sufficient data were excluded.^8,36–39^ Using the review of the literature data, the frequency of large-vessel inflammation in GCA patients was determined at 87% and is much higher than the frequency usually described using Computed Tomography Angiography (CTA). The presence of aortitis in CTA varies between 20% and 65% in the literature.^40,41^ However, in the studies included in this meta-analysis, no comparison was made between FDG vascular uptake and findings of CTA. Consequently, the superiority of FDG-PET over CTA for the diagnosis of large-vessel inflammation in GCA cannot be determined so far. Other reasons could be argued to explain the high rate of FGD vascular uptake in GCA patients. The presence of atherosclerosis and increasing rate of vascular uptake with age could partly overestimate the rate of large-vessel inflammation in older patients.^42^ In the study of Tatsumi et al,^42^ including 85 consecutive patients with neoplasms, FDG uptake was commonly observed in the thoracic aortic wall, particularly in older patients, with hyperlipidemia or cardiovascular disease. Another point of discussion is the choice of PET criteria to define the presence of vascular inflammation, as no reliable parameter has been consensually defined yet. Our systematic review shows that the visual vascular uptake equal to or higher than liver uptake was observed in 84% of LVV versus 18% controls (Table 2) and could constitute the PET criteria for the diagnosis of LVV. It is also important to note that the wall distribution of FDG uptake was not considered in the literature studies, and usually in LVV the lesions are circumferential while the atherosclerotic lesion is quite limited.

The second issue is the diagnosis of underlying vascular inflammation in TA. The definition of disease activity in TA is quite challenging, as radiological progression could be observed in patients without acute-phase reactants. Our study shows that PET allows a reliable assessment of inflammatory activity in TA, with a Se and Sp at 87% and 73%, respectively, and with a moderate increase in positive LR (4.2). Sp increased at 84% when considering patients with conventional disease activity assessment, that is NIH scale. Individual data analysis in TA patients showed that visual and semiquantitative analysis were helpful in the assessment of disease activity, as FDG uptake correlated with the presence of markers of inflammation. Yet, a moderate uptake was observed in a significant number of patients with inactive TA without any markers of inflammation (7/12, 58%, Table 4). This point is particularly important as it is well-established that disease activity is particularly difficult to ascertain, and previously postmortem histological studies showed a significant rate of vascular inflammation in patients considered to be having inactive TA disease.^43^ Indeed, active vascular inflammation could be found in patients with neither clinical nor biological activities, and imaging tools mostly consider the progression or new arterial lesions, but poorly correlate to the vascular inflammation. The assessment of vascular inflammation is a crucial issue, as it is correlated with arterial progression, and development of stenosis and thrombosis. Other prospective studies are needed to determine whether the presence of FDG uptake in patients without clinically active disease could be correlated to the arterial progression, and thus vascular complications.

Our study has several biases, which could limit definitive conclusions. The main limitation of this meta-analysis is the different PET technologies used across studies, with about 50% (12/21) of studies using a nonhybrid technology. In addition, different parameters were used to define the presence of vascular inflammation, and this point underlines the need to standardize the PET criteria for LVV. Larger studies are needed to define accurate parameters to analyze the vascular inflammation by PET (visual analysis or semiquantitative or both), and define the threshold to ascertain the vascular inflammation. Other PET criteria, such as the pattern of FDG uptake (a circumferential uptake could be more consistent with vascular inflammation of LVV than atherosclerotic lesions), should be considered in order to increase the robustness of PET analysis. Besides, the different conditions of the controls (infectious, inflammatory process, or malignancy) are another issue of this meta-analysis, and large-vessel inflammatory process cannot be absolutely excluded in these patients. However, it should be noticed that none of the controls met the LVV classification criteria. Finally, about one-third of patients were under corticosteroid and/or immunosuppressive treatment at the time of PET study, which could bias the performance of PET in these patients.

There are remaining issues that should be addressed in future studies in order to increase the accuracy of FDG-PET in staging LVV, particularly the study of the FDG uptake patterns of extension and distribution in different arterial areas, as well as the correlation of FDG uptake with the CTA arterial wall thickening.

## CONCLUSIONS

FDG-PET appears to be a suitable modality for the detection of large-vessel inflammation in both GCA patients and TA patients. FDG-PET demonstrated a good performance in the diagnosis of large-vessel inflammation in GCA patients, with a pooled Se at 90% and a pooled Sp at 98%. In TA patients, FDG-PET showed an overall good accuracy for the assessment of disease activity with pooled Se and Sp at 84%. However, the presence of a significant vascular uptake can be observed in TA patients without markers of activity, and further studies are needed to determine whether it reflects infraclinical inflammation and correlates with arterial progression and vascular complications.
